# Challenges of drug-resistant malaria

**DOI:** 10.1051/parasite/2014059

**Published:** 2014-11-18

**Authors:** Shweta Sinha, Bikash Medhi, Rakesh Sehgal

**Affiliations:** 1 Department of Medical Parasitology, Postgraduate Institute of Medical Education and Research Chandigarh 160012 India; 2 Department of Pharmacology, Postgraduate Institute of Medical Education and Research Chandigarh 160012 India

**Keywords:** Malaria, Drug Resistance, Nanotechnology, RNAi, Stem cell, Peptides

## Abstract

Over the past six decades, the drug resistance of *Plasmodium falciparum* has become an issue of utmost concern. Despite the remarkable progress that has been made in recent years in reducing the mortality rate to about 30% with the scaling-up of vector control, introduction of artemisinin-based combination therapies and other malaria control strategies, the confirmation of artemisinin resistance on the Cambodia–Thailand border threatened all the previous success. This review addresses the global scenario of antimalarial resistance and factors associated with it, with the main emphasis on futuristic approaches like nanotechnology and stem cell therapy that may impede resistant malaria, along with novel medications which are preparing to enter the global antimalarial market. These novel studies are likely to escalate over the coming years and will hopefully help to reduce the burden of malaria.

## Abstract (fr)

Au cours des six dernières décennies, la résistance aux médicaments de *Plasmodium falciparum* est devenue une question extrêmement préoccupante. Malgré des progrès remarquables accomplis ces dernières années pour réduire le taux de mortalité à environ 30 %, avec l’intensification de la lutte antivectorielle, l’introduction des thérapies combinées basées sur l’artémisinine et d’autres stratégies de lutte contre le paludisme, la confirmation de la résistance à l’artémisinine sur la frontière Cambodge-Thaïlande a menacé tous les succès précédents. Cette synthèse porte sur le scénario global de diverses résistances antipaludiques et les facteurs qui y sont associés, en soulignant les approches futuristes comme la nanotechnologie et la thérapie par les cellules souches, qui peuvent entraver le paludisme résistant, et les nouveaux médicaments qui vont bientôt entrer sur le marché antipaludéen mondial. Ces nouvelles études vont s’intensifier au cours des prochaines années et, nous l’espérons, contribuer à réduire la charge du paludisme.

## Introduction

Malaria has been one of the most extensively studied parasitic infectious diseases for millennia. In 2012, there were around 627,000 malaria deaths worldwide, 90% of which were in the African Region, followed by Southeast Asia (7%) and the Eastern Mediterranean (3%). About 482,000 malaria deaths were estimated to occur in children under 5 years of age, constituting 77% of the global total. Most of these deaths were due to *Plasmodium falciparum.* However, *Plasmodium vivax* is now increasingly recognized as a cause of severe malaria and death [139]. For decades, drug resistance has been one of the greatest obstacles in fighting malaria. To date, drug resistance has been reported in three of the five *Plasmodium* species that is, *P. falciparum*, *P. vivax* and in *P. malariae* which are the causative agents for human malaria [45]. Drug resistance was initially outlined by WHO in 1967 as the ability of a parasite strain to survive or reproduce regardless of the administration and absorption of a drug when it is given in doses that are equal to or higher than those usually recommended but within the tolerance range of the given subject [137]. This was later modified by Bruce-Chwatt et al. [20] to include “the amount of the drug which is active against a given parasite must be able to gain access to the parasite or the infected erythrocyte for the length of the time necessary for its natural reaction”. Drug resistance usually leads to a delay or failure to clear asexual parasites from the peripheral blood that eventually enable production of gametocytes which are responsible for transmission of the resistant genotype. After the official recommendation by the WHO in 2001 [132] for use of artemisinin-based combination therapies (ACTs) as the first-line treatment of *P. falciparum* malaria, it was seen after 2005 that there was a substantial decline in outbreak of this disease [134]. However, parasites that are drug resistant to artemisinin and its derivatives have recently emerged in various parts of Southeast Asia, which threaten, all prior success of malaria control strategies, treatment and elimination efforts [30, 38]. At present, current antimalarial drugs act on a limited number of biological targets [124]. Therefore, the next challenge is to identify new classes of drugs that will attack novel molecular targets, with sufficient therapeutic lifespans that will not be compromised by the rapid development of resistance, and to develop novel technologies, that will effectively clear the parasite with maximum precision, thus minimising the risk of drug resistance [25]. This review summarises current scenarios, along with existing therapies and novel on-going approaches to curb drug-resistant malaria.

## Current scenario of drug resistance

Of the various antimalarial drugs available, the aminoquinoline chloroquine was the agent of choice for many decades because of its safety, efficacy and affordability. However, parasite resistance to this drug was initially observed in Thailand in 1957 and then on the border of Colombia and Venezuela in 1959. By the late 1970s, resistance reached East Africa and by the mid-1980s had become a major problem in several areas of the continent [128]. At present, chloroquine remains effective only in some parts of Central America, where clinical studies have confirmed it as an effective drug [69]. However, recent data on the prevalence of chloroquine-resistant genotypes in these areas present an alarming situation for health officials [36]. Amodiaquine has been observed to be more effective than chloroquine mainly in areas of persistent chloroquine resistance. As a result, amodiaquine in combination with artesunate was adopted as the first-line treatment by several countries. Parasite strains that are highly resistant to amodiaquine have however been reported in Tanzania, which may additionally compromise the use of artesunate-amodiaquine in Africa [106]. Another antimalarial, sulfadoxine-pyrimethamine, has been widely used by several countries to treat chloroquine-resistant malaria. Nonetheless, the treatment failure rate of this combination has been found to be low in several countries of South America and Central and Middle East Asia, as compared to the failure rate in eastern Africa (52.8%) [45]. Presently, resistance to mefloquine continues to be a concern in the Greater Mekong sub-region, in particular in Thailand and Cambodia, where artesunate-mefloquine is still used as first-line treatment [108]. In order to maximise the effectiveness of artemisinin and its derivatives and to protect them from the development of resistance, WHO has repeatedly recommended that they can be combined with other drugs that have different mechanisms of action and longer half-lives. As a result, five combinations are currently recommended: artemether-lumefantrine, artesunate-amodiaquine, artesunate-mefloquine, artesunate-sulfadoxine-pyrimethamine and dihydroartemisinin-piperaquine (WHO, 2010) [138]. However, remarkable failure rates of these combinations have been observed in several African countries where resistance to one drug has been previously encountered, like in the case of artemether-lumefantrine. Artemether-lumefantrine remains highly effective in most parts of the world, with the exception of Cambodia. This combination mostly shows failure rates less than 10% [45]. However, resistance to most of these combinations will probably lead to a global epidemic outbreak of malaria. To overcome this concern, GlaxoSmithKline (GSK) along with the PATH Malaria Vaccine Initiative (MVI), with a grant from the Bill & Melinda Gates Foundation, have developed RTS, S/AS01, the most advanced candidate, which has proven its protective efficacy in children with a range of 30–50% [2, 3] and is believed to represent the first-generation malaria vaccine (WHO recommendation expected by 2015). Nevertheless, with data from Phase III trials indicating that the leading malaria vaccine candidate, RTS, S, has limited efficacy, it is important to consider new approaches for the development of a vaccine that is capable of inducing long-term protection [112].

## Reasons leading to antimalarial resistance

**Figure 1.**
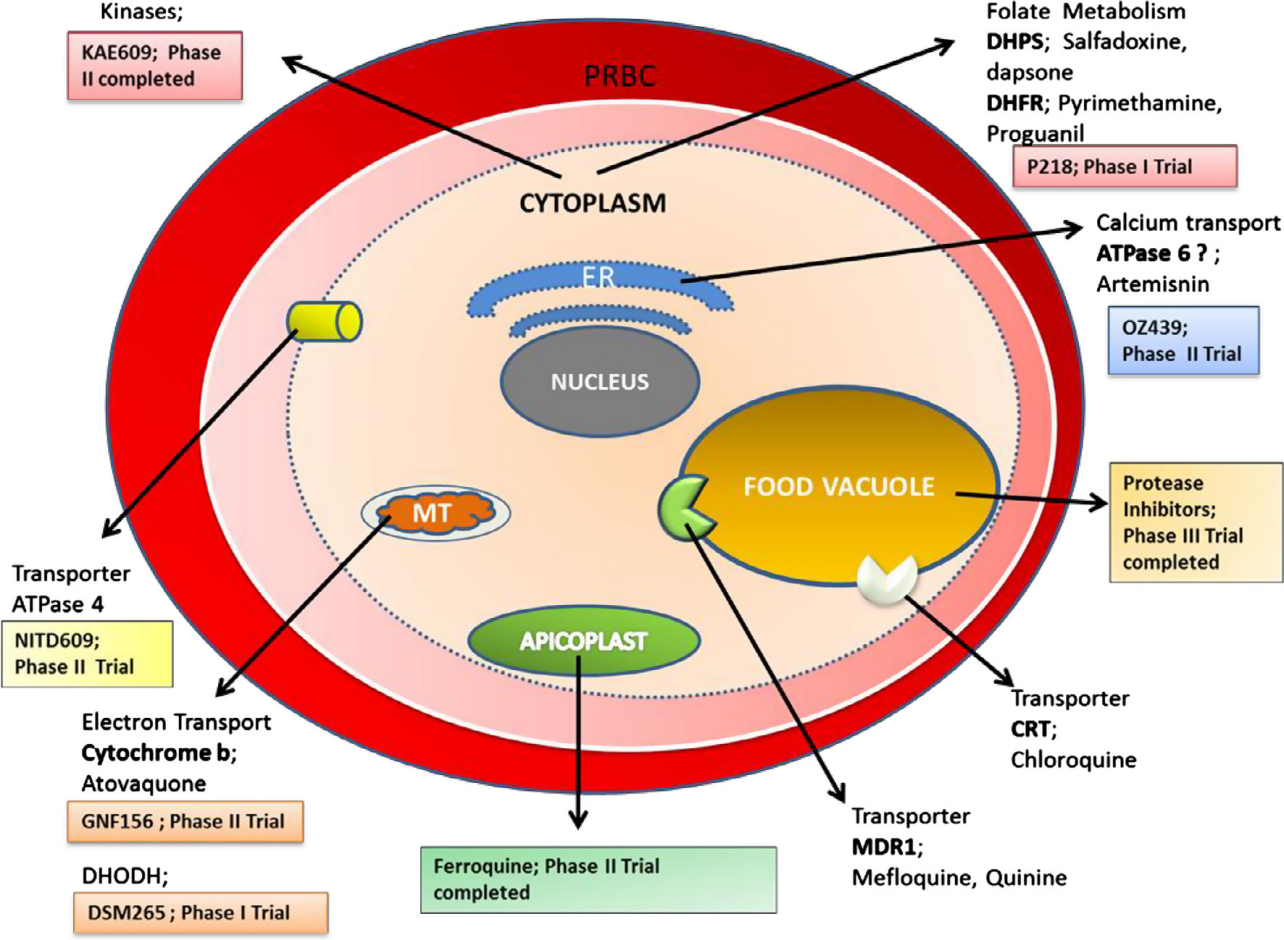
Different proteins present inside the parasitic organelle that contribute to drug resistance in malaria under selective drug pressure and new drugs in development, targeting the same pathway to rescue resistance. PRBC: parasitized red blood cell, ER: endoplasmic reticulum, MT: mitochondria, DHPS: dihydropterate synthetase, DHFR: dihydrofolate reductase, ATPase6: sacro/endoplasmic reticulum calcium dependent ATPase orthologue, CRT: chloroquine resistance transporter, MDR1: multidrug resistance.

Various factors lead to the occurrence and massive spread of resistance. Genetic mutations that confer antimalarial drug resistance mostly occur in nature and are independent of drug effect and are considered spontaneous mutations. The onset of resistance is thought to occur in two phases. In the first phase, an initial genetic event produces a resistant mutant (*de novo* mutation) in which a new genetic trait gives the parasite a survival advantage against the drug. In the second phase, the resistant parasites are then selected and start to multiply, which finally ends with a parasite population no longer being susceptible to treatment. For a few drugs, to confer resistance there is only involvement of single point mutation, however for various other drugs multiple site mutation is required. The acquired mutations allow the survival or reproduction of the resistant parasite whereas drug pressure will eliminate susceptible ones [90]. Antimalarial drug resistance typically arises when there are spontaneous mutations that are selected by different concentrations of anti-malarial drug that impart deferential inhibition to distinct genetic parasite types, i.e. the drug concentrations are sufficient to reduce the susceptible parasite population, but can either not inhibit multiplication or cause less inhibition of the mutants [92]. Drug resistance to several antimalarials is sometimes either due to changes in drug accumulation or efflux mechanisms (chloroquine, amodiaquine, quinine, halofantrine, mefloquine resistance) or due to decreased affinity of the drug target which may result from point mutations in the respective genes that encode these targets (pyrimethamine, cycloguanil, sulphonamide, atovaquone, artemisinin resistance) [6, 42, 75, 113, 125], shown in Figure 1.

A limited number of genes involved or potentially involved in *P. falciparum* antimalarial drug resistance have been identified: the genes encoding dihydropteroate synthase (Pfdhps), dihydrofolate reductase (Pfdhfr), the chloroquine resistance transporter (Pfcrt), the multidrug resistance 1 protein (Pfmdr1), Na^+^/H^+^ exchanger (Pfnhe-1) and cytochrome b, shown in Table 1.

**Table 1.** First reported resistance to antimalarial drugs and molecular markers for drug resistance.

Antimalarial drug	Introduction date	First reported resistance	Molecular marker	References
Quinine	1632	1910	pfnhe: microsatellite ms4670	[40, 53, 87, 91]
Chloroquine	1945	1957	crt: C72S, M74I, N75D/E, K76T, A220S, Q271E mdr1: N86Y, Y184F, S1034C, N1042D, D1246Y	[19, 29, 33, 128]
Proguanil	1948	1949	dhfr: A16V, S108T or N51I, C59R, S108N, I164L	[49, 54, 91]
Sulfadoxine + Pyrimethamine	1967	1967	dhps: S436A/F, A437G, K540E, A581G, A613S/T dhfr: N51I, C59R, S108N, I164L or C50R, N51I, S108N, I164L	[16, 91]
Mefloquine	1977	1982	Deamplification of Pfmdr1 copy	[55, 88]
Halofantrine	1988	1993	Changes in Pfmdr1 copy number	[18, 123, 129]
Atovaquone	1996	1996	cyt b: Y268S/N	[60, 70, 135]
Artemisinin	1971	1980	Amplification of Pfmdr1 copy numbers, Mutation of PfATPase6 and Pfubp-1. Recently, mutation in K13-Propeller Domain has been confirmed.	[35, 75, 103, 113, 133]
Artesunate	1975	2008	NA	[86]
Artesunate + Mefloquine	2000	2009	Deamplification of Pfmdr1 copy	[31, 73, 103]

Drug resistance is complicated by cross-resistance, which mostly occurs among the groups of drugs which belong to a similar chemical family or which have the same mechanism of action, for example, development of cross-resistance between halofantrine and mefloquine [101]. Furthermore, multiple drug resistance of *P. falciparum* has been seen when the parasite is resistant to more than two operational antimalarial compounds of different chemical classes and modes of action.

Recently, the role of pharmacokinetics in determining antimalarial efficacy and in promoting the emergence and spread of drug resistance has gained far more attention [10]. In the past, plasma levels of drugs were rarely measured, so it was thought that all episodes leading to clinical treatment failure were due to inherent parasite resistance. The dose selected is usually the lowest dose that achieves a good response so as to minimise adverse effects. However, during expansion of resistance, it has been found that relatively low amounts of drug allow remarkable spread of resistant parasites because the therapeutic level that is needed to clear partially resistant parasites is usually higher than the level which is required to eliminate the fully susceptible ones [11]. Therefore, incomplete understanding of pharmacokinetic factors may shorten the useful life of antimalarial drugs and may hasten the spread of resistance.

**Figure 2.**
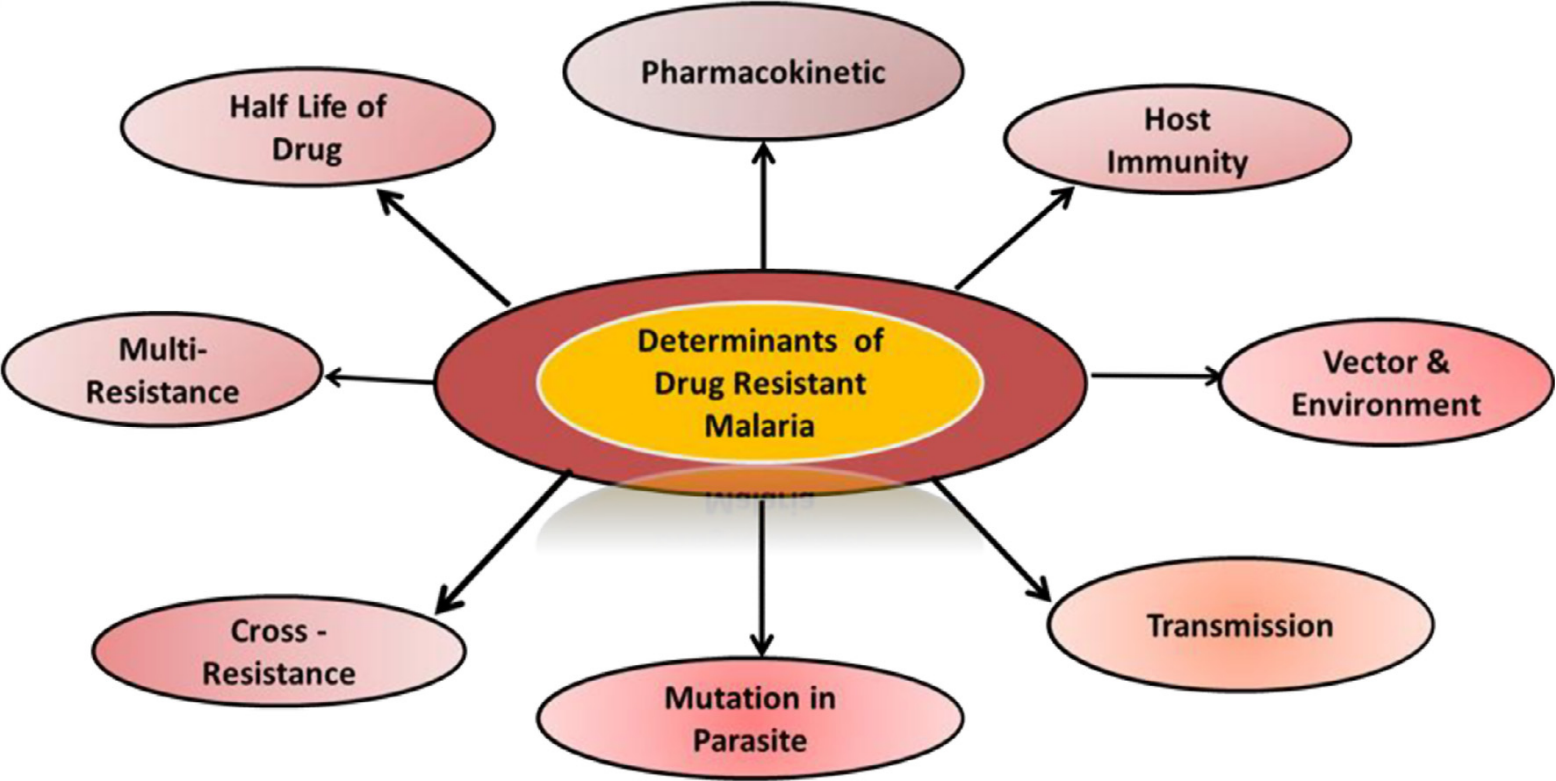
Different parameters that contribute to antimalarial resistance.

The subsequent spread of resistant mutant malaria parasites is facilitated by administration of drugs with longer elimination phases. The remaining antimalarial activity which persists during the post-treatment period acts as a “selective filter”, that found to be preventive in case of infection by sensitive parasites, but enables infection by resistant parasites. Drugs like chloroquine, mefloquine and piperaquine, which persist for longer durations in the blood plasma, provide a selective filter long after administration has ended [140]. The longer the terminal elimination half-life of the drug, the greater is the chance that any freshly acquired parasite can encounter partially effective drug concentrations [51, 126, 127]. The duration of the terminal elimination half-life is therefore a vital determinant of the propensity for an antimalarial drug to become ineffective because of the development of resistance. The prolonged presence of drugs like mefloquine, piperaquine and chloroquine in the host’s blood provides a lengthy exposure time during which resistant parasites may be selected [131] (Fig. 2).

The host immune response to malaria infection likely influences the speed of spread of drug resistance and the extent to which drug resistance translates into clinical drug failure [52]. Host defence contains a major antiparasitic impact, and any drug-resistant mutant *Plasmodium* that generated spontaneously should contend not only with the antimalarial drug concentrations but also with host immunity that kills parasites irrespective of their antimalarial resistance and reduces the likelihood of parasite survival (independently of drugs) at almost all stages of the transmission cycle. Immunity in case of *Plasmodium* infection acts by non-specifically eliminating erythrocytic-stage parasites which includes rare *de novo* resistant mutants, and found to improve curative rates, even with the failing drugs, hence diminishing the comparative transmission advantage taken by the resistant parasites. If a resistant mutant survives the initial drug regimens and is able to proliferate, this will then often result in sufficient gametocytes which finally increase the disease transmission rate. However this frequency of transmission can be decreased if there is immunity against the asexual stage (which reduces the multiplication rate and lowers the density at which the infection is controlled) and to the sexual stage.

Epidemiological studies have implicated low-transmission settings as the primary origin of drug resistance [102]. This is most likely attributable to the fact that in areas of low-transmission intensity, most of the malaria infections are symptomatic and so, proportionately lots of people receive treatment, which ultimately generates higher chances for selection. However, there is less chance of emergence of drug resistance in areas with high-transmission intensity primarily because most malaria infections are asymptomatic and infections are acquired repeatedly throughout life. Also, in high-transmission areas, malaria-experienced populations slowly acquire partial immunity (“premunition”), and therefore the infection is controlled, sometimes at levels below those that cause people to develop symptoms.

Finally, vector and environmental factors may influence the proliferation of resistant parasites [129]. It is likely that drug-resistant parasites are less fit compared to their wild type. As such, there is a possibility of disappearance of resistant parasites by removing the drug pressure [8]. Recently, Lewis et al. studied the re-emergence of a chloroquine-sensitive strain in a wild population by demonstrating that development of chloroquine resistance is mainly due to a defect in the ability of the parasite to degrade haemoglobin, which inhibits active reproduction compared to sensitive ones [67].

## Novel on-going approaches to impede drug resistant malaria

The treatment regimens of malaria are directly correlated to parasite drug resistance and dictated by government political strategies of prevention and control of morbidity and mortality due to the disease [9]. Considering the few number of new drugs or innovative antimalarial medicines approved since 1990, the search for more potent and less toxic antimalarials, the development of a successful vaccine, antimalarial peptides and the design of nanotechnology-based delivery systems applied to drugs and antigens and other approaches like RNA interference (RNAi) and stem cell therapy, are likely to be the main strategies in combating this disease. However, the main drawbacks associated with conventional malaria chemotherapy are the development of single or multiple drug resistance and the non-specific targeting to intracellular parasites which results in high dose requirements and subsequent intolerable toxicity that provides a new vision to apply novel approaches in disease treatment.

### RNA interference (RNAi)

RNA interference (RNAi) is a method of interrupting gene expression that acts as a post-transcriptional event specifically degrading targeted mRNA that results in decreased synthesis of specific proteins [41]. RNAi is now emerging as a powerful technology with vast applications for genomics, elucidation of molecular signalling pathways, and target identification in drug discovery. Presently, RNAi studies reveal the clinical potential of small interfering RNAs (siRNAs) in metabolic diseases, AIDS, cancer, malaria, dental diseases, neurodegenerative disorders and various other illnesses. Recent studies have shown that the tiny RNA molecules, either endogenously made as microRNAs (miRNAs) or exogenously administered as synthetic double-stranded RNAs (dsRNAs) could efficiently activate a selective gene in a sequence specific manner despite silencing it [96]. The recent discovery of RNAi and its possible adaptation to mosquitoes is now contributing as a crucial tool for understanding vector-parasite interactions as well as providing insight to analyse different aspects of mosquito biology that could influence vectorial capacity. At present, two RNAi approaches have been well-established in mosquitoes. Firstly, transient gene silencing achieved by direct delivery of double-stranded RNA, and secondly, steady and stable expression of hairpin RNAs from the transgenes that integrated in the genome [21]. Few studies has been reported about the role of RNAi in *Plasmodium* and then taken as a tool to understand their genetic function. Although these studies [72, 74] reveal the down-regulation of gene expression after application of dsRNA targeting specific proteins in *P. falciparum*, they were unable to prove that these introduced dsRNA were ultimately processed to true siRNA inside *Plasmodium*. Mohammed et al. also reported specific down-regulation of *P. berghei* cysteine protease berghepain when targeted with siRNAs in *Plasmodium berghei*-infected mice and resulted in only about 0.01% of the siRNA which are being internalised inside the parasite that leads to 40–50% reduction in berghepain mRNA levels. However, the experiment did not alter the level of parasitaemia in infected mice treated with siRNA. In addition, others have reported the successful application of RNAi for silencing genes at the blood stage of *Plasmodium* [77]. In this context, various experiments have been carried out by employing the technique of electroporation to introduce long dsRNAs within infected erythrocytes. Kumar et al. successfully applied siRNA to confirm the essential role of *P. falciparum* serine/threonine phosphatase (PP) in *Plasmodium* growth as it was found to be expressed all along the erythrocytic stages [63]. Gissot et al. performed gene silencing experiments by using pfmyb1 double-stranded RNA (dsRNA) to interfere with the cognate messenger expression which results in 40% reduction in parasite growth as compared to untreated culture [44]. *P. falciparum* signal peptidase was also found to be essential for the intra-erythrocytic growth of the parasite, as implication of PfSP21 dsRNA specifically leads to inhibition in the growth of *P. falciparum* [118]. Tuteja and Pradhan elucidated the application of RNAi by targeting *P. falciparum* translation initiation complex eIF4F with the specific dsRNA corresponding to each complex for revealing the importance of these initiation complexes in parasite multiplication, and shows approximate 45% reduction in parasite growth [119]. In a recent study, *P. falciparum* UvrD helicase revealed that N-terminal UvrD (PfUDN) dsRNA shows inhibition in the growth of the parasite particularly during earlier phase of schizont development. The study reveals that the growth of parasite was inhibited by approximately 40% by adding PfUDN dsRNA in culture [114]. Another study observed transcriptional down-regulation of the hypusinated form of either eIF-5A or DHS upon transfection of *P. berghei* ANKA merozoites with eIF-5A-shRNA and DHS-shRNA, respectively, as hypusination of eIF-5A is important for cell proliferation of the parasite. This study provided evidence for a noncanonical RNAi-related pathway [109]. However, other studies have revealed the absence of RNAi genes [13]. Taken together, the presence of RNAi in *Plasmodium* is still quite contradictory. The experimental identification and validation of the *P. falciparum* small antisense nonprotein-coding RNA (npcRNA) transcriptome may provide an alternative to the classical RNAi pathway [97].

RNAi technology is one of the most significant advances to elucidate the genetic function of an organism, but its clinical implication in malarial parasites is limited. However, two major organelles, having their own genomes, i.e. *Plasmodial* apicoplast and mitochondria, are consider as a potent drug target with respect to their function. These organelles might be explored clinically in coming decades for their particular function in parasite growth by applying RNAi technology [80]. Looking into *Plasmodial* apicoplast reveals homology with respect to plant and algal chloroplast that share various metabolic pathways found to be unique to plants like biosynthesis of isoprenoids, fatty acid and heme synthesis. But at the same time, *Plasmodial* apicoplast has lost its photosynthetic function. This apicoplast is also involved in housekeeping functions which are found to be the same as bacterial housekeeping. Therefore, non-existence of these pathways in humans suggests apicoplast as a major drug target. Moreover, the functional activity of apicoplast like replication, transcription and translation suggests it as an immense and effective target for various anti-apicomplexan drugs [71]. Studies have inferred the uses of reverse genetic techniques to get a clear picture of apicoplast function along with its various processes that helps in parasite growth and survival [47]. Furthermore, *Plasmodial* mitochondria are the second major organelle regulating various metabolic processes all along the *Plasmodium* life cycle [85]. The active electron transport chain of *Plasmodium* contains distinct dehydrogenases compared to human mitochondria so the driving metabolic energy is also quite different from the mammalian host [117] which shows *Plasmodial* mitochondria as a valuable drug target and it can also be exploited for applying RNAi clinically.

There are numerous advantages of RNAi for studying gene function in *P. falciparum*: the assay can be conducted in a few days compared with months required for gene inactivation, multiple genes can be analysed simultaneously for genome screening purposes, the cost is considerably less than the synthesis of modified antisense oligonucleotides, and the transient nature of the assay may be an advantage for investigating essential genes. Disadvantages with the current methodology include the dependence on the electroporation efficiency and the lack of a marker phenotype following manipulation of this organism. Both areas of optimisation are currently under investigation. Moreover, effective delivery of RNAi is the biggest concern in *Plasmodium* as it has to cross the erythrocyte membrane, parasitophorous vacuolar membrane, parasite cytoplasm membrane and the parasite nuclear membrane in order to reach the *Plasmodium* nuclei [141].

Techniques used for genetic interference in *Plasmodium* are found to be refractory especially for the genes that are involved in the blood stage development of the parasite. So, the transformation of an RNAi deficient *Plasmodium* into RNAi possessing one with the lowest set of RNAi transgene machinery might be the foremost challenge for implication of RNAi technology [32]. Thus, more studies will be needed to elucidate the mechanisms of gene silencing observed in *Plasmodium* and to assess the therapeutic potential of RNAi in this important parasite. In future, practical implications of RNAi in gene silencing will provide us powerful means for developing novel therapeutics [120].

### Nanotechnology

The development of drug resistance by malaria parasites may also be due to the use of ineffective pharmaceutical dosage forms of antimalarials. In pharmaceutical sciences, nanotechnology has made dissolution rates remarkably faster and higher, increased the bioavailability of many drugs, and improved the stability of sensitive agents. To our current concern, nanosized carriers are now receiving special attention with the aim of minimising the side effects that arises from conventional drug therapy, like inappropriate bioavailability and least selectivity of drugs. These nanocarriers have now been implicated for malaria diagnosis [110, 136] and treatment [43] and in vaccine formulation [4]. Malaria parasites frequently develop drug resistance due to the administration of low drug concentrations in the presence of a high parasitic count [83]. Furthermore, nanotechnology has the potential to restore the use of old and toxic drugs by modifying their bio-distribution and reducing toxicity [43]. This advantage is particularly important in malaria therapy, since the development of new dosage forms for delivering drugs to parasite infected cells is urgently needed, especially for the antimalarials in clinical use. Nanocarriers may not only allow the use of potentially toxic antimalarials [26], but also increase the efficacy of immune response in vaccine formulations [89].

Several nanosized delivery systems have previously been proved in terms of their effectiveness in experimental models for the treatment and prophylaxis of malaria. For example, a rapid test for malaria diagnosis was developed by the Udomsangpetch group, based on agglutination of sensitive polystyrene particles [94], in order to overcome prior limitations which are associated with the high cost of currently available rapid diagnostic kits. This test includes aggregation of nanoparticles with antigen or antibody-called latex-antigen (or antibody) conjugates under *in vitro* conditions, in the presence of malaria specific antibody (or antigen). The assay was successfully evaluated for *P. falciparum* at an outpatient malaria clinic (Mae Sot, Thailand) and claimed to be the quickest and easiest test that can be performed in the field. *Plasmodium* throughout its intra-erythrocytic phase modifies the host RBC plasma membrane, which has made lipid-based nanocarrier as the most promising carrier for targeting the infected RBCs [26]. This includes encapsulated liposomes which have been used for targeted delivery of antimalarials *in vivo* [95]. Liposomal nanovessels containing amino acid sequences of *P. berghei* have been used to target the hepatocyte stage of *Plasmodium* [100]. Others formulated PEGylated liposomes containing artemisinin, which is mainly a long circulating vesicle representing an efficient nanocarrier that can be used therapeutically in parasitic infection as well as in tumours [56]. However, despite these appreciable results, liposomal-based delivery was not adopted in disease rescue programmes mainly due to its non-selectivity towards parasitised RBCs. In this context, Urbán et al. [121] developed a nanovector for targeted delivery of antimalarials to human parasitized RBCs. At the same time, these lipid-based nano-delivery systems provide a frame to re-formulate existing and toxic antimalarials for achieving better and appreciable pharmacokinetic profiles, and biodistribution, along with appropriate targetability [57]. To this end, transferrin-modified-artemether lipid nanospheres have also been developed as targeted drug delivery systems against tumour cell lines [37]. Significant results shows its might be beneficial in targeting parasitised RBCs. Earlier, Tayade and Nagarsenker formulated microemulsions of artemether which have 1.5 times better antimalarial activity than the marketed one, Larither^®^, which was mainly due to quick release of drug from their formulation [115].

In addition, the most important property of a nanocarrier in the context of malaria is its ability to remain for a longer time in the blood stream in order to improve the interaction with infected RBCs and parasite membranes [78]. Furthermore, other features are protection against unstable drugs, properties of cell adhesion, as well as the potential to be modified at the surface after being conjugated by specific ligands [59]. Remarkably, during treatment of cerebral malaria, most of these potential benefits can be achieved by colloidal nanocarriers that can be administered intravenously. In case of uncomplicated malaria, the non-parenteral routes are mainly preferred, but this reduces the spectrum of possibilities in terms of the use of drug nanocarriers. Various strategies have been made to make it possible to implement this technology to curb malaria [23, 26, 50, 66]. There are two main strategies for targeting antimalarial drugs to the infected erythrocytes and occasionally the hepatocytes using nanocarriers by the intravenous route: passive and active targeting. Passive targeting is mostly achieved using conventional nanocarriers (e.g. liposomes, hydrophobic polymeric nanoparticles) [12] or surface-modified long-circulating nanocarriers (e.g. PEGylated) [48, 65]. On the contrary, active targeting is achieved by means of nanocarriers surface-modified with specific ligands such as carbohydrates, proteins, peptides or antibodies [12]. Recently, polymer-based nanoaggregates, i.e. polyamidoamine (PAA)-derived polymer, have been used for administering antimalarials into pRBCs. However, after encapsulation, *in vitro* efficacy of the drugs was found to be moderate [122]. Afterwards, Movellan et al. developed Janus dendrimer based on 2,2-bis(hydroxymethyl)propionic acid (bis-MPA) monomers for encapsulating two antimalarial, i.e. chloroquine and primaquine and their results show significant reduction in *in vitro* IC50 compared to free drug. Also, encapsulation of primaquine was found to be promising as compared to free primaquine which causes haemolysis in patients having deficiency of glucose-6-phosphate dehydrogenase (G6PD) [79]. The wide range of modulations of the surface properties of these nanometric carriers aimed at improving antimalarial selectivity in the recently-discovered parasite targets has been little exploited to date. From this study it emerged that nanotechnology applied to malaria therapy is a domain that is still in its infancy [107].

Apart from nanocarriers, nano/microfluidic technologies are also emerging as methods that could address the challenges imposed by other conventional diagnostic devices [24, 81]. These approaches enable real-time monitoring of infectious diseases from a small volume of bodily fluids [35], and can be used to integrate various assays into a single device [28, 64, 111, 130], and have the ability to deliver each sample to specific reaction chambers in a systematic manner [58]. Among these technologies, nanofluidics have been highlighted by the recent advent of nanoscience and nanotechnology since the rise of microfluidics in the 1990s. Generally, nanofluidics can be defined as the field of study in fluid flow in and around nanoscale objects [34]. For example, the RDT strip chip can detect proteins derived from the blood of malaria parasites in a microfluidic format and can also be realised as commercialized products. This chip enables the generation of a series of visible lines to indicate the presence of specific antigens in blood that are clearly visible to the naked eye when antibody is accumulated at the test line. Rathod et al. developed microfluidic channels to study malaria pathogenesis related complex interactions between host cell ligands and parasitised erythrocytes [5]. Since the microfluidic channels successfully mimic the sizes and shapes of capillary blood vessels, they could observe host-parasite interaction and malaria-infected red blood cells in a capillary environment. The malaria diagnostic device is inexpensive and handheld for on-site analysis of patient samples and only requires microliter sample volume. Therefore, it has the potential to be widely used at field sites for more accurate malaria diagnosis. Nanotechnology systems may therefore afford a better therapeutic outcome by targeting drugs specifically to their site of action.

### Stem cells

Stem cells are unspecialised cells found in embryos during the blastocyst stage and in various tissues of adults. They have the typical characteristic of dividing mitotically in order to self-renew and differentiate into various types of cells under appropriate conditions for each specific function. They also serve as cell reservoirs for fulfilling the purpose of repair of damaged tissues inside the body. Recent studies suggests that stem cells, especially the mesenchymal stem cells, have immuno-modulatory characteristics and due to this property many trials are being conducted by transplanting these mesenchymal stem cells in disease conditions which are thought to arise from immunological abuse.

Severe destruction of red blood cells causes anaemia, thus posing pressure on bone marrow to meet the requirements of myeloid cells. Scientists from the National Institute for Medical Research, UK, have identified an atypical progenitor cell from malaria infected mice which can give rise to a lineage of cells capable of fighting this disease [14]. Transplantation of these specific cells into mice with severe malaria was found to help these infected mice in recovering from the disease. Other reports also support stem cell therapy for malaria treatment [105]. Manipulation in stem cells can also produce erythrocytes with modified haemoglobin variants that are associated with protection from malaria. Thakur et al. [99] identified recruitment of MSCs as a novel host protective mechanism adopted by the host to combat malaria by modulating Treg-cell responses. A massive accumulation of Sca-1+CD44+CD29+CD34− cells (where Sca-1 is stem cell antigen-1), a phenotype consistent with mesenchymal stem cells (MSCs) but not conventional stem cells, was found in this study. Infusion of purified MSCs from infected animals to naive animals dramatically protected against infection by *Plasmodium berghei* (Pb). Furthermore, prior infusion of MSCs from infected mice prevented splenomegaly, infiltration of NKT cells, and haemozoin accumulation. This was accompanied by a profound reduction in inhibitory cytokines such as IL-10 and up-regulation of inflammatory cytokines such as IL-12 and IL-1*β*. In addition, these animals had reduced levels of Treg cells that were able to dampen antigen specific protective immune responses. Taken together, the study identified accumulation of MSCs as a novel host response to combat malaria by inhibiting haemozoin and anti-inflammatory cytokine production, and by reducing Treg-cell accumulation in the spleen. In addition, multipotent haematopoietic stem cells were reported to play an important role in the host’s defence mechanisms against *Plasmodium berghei* infection [7]. Based on these studies, it is believed that although stem cell therapy is at initial stages, it will soon be a real therapeutic option for various parasitic diseases and with continuous effort along with vast knowledge of the present subject will lead to new experimental models, appropriate type and number of stem cells, route of administration and similar disease conditions that will possibly be beneficial for the treatment of the patient with a parasitic infection [142]. Therefore, approaches may differ from disease to disease but stem cells are always in focus to treat several diseases including malaria.

## Miscellaneous approaches

### Peptides

Besides these novel approaches, peptides that has been isolated from various natural sources like plant, fungi and bacteria or derived synthetically are often widely explored novel molecules that have large chemotherapeutic potential and can serve various diseases including malaria [62]. They are basically secondary metabolites displaying huge amounts of heterogeneity in their primary as well as secondary structures. However, they share some common features that reveal their cytotoxic activity, such as amphipathic along with net positive charge [17]. Although their exact mechanism of action has not been completely studied, most are considered to have cytocidal effects by disintegrating the membrane structure [15].

In addition to various antimalarial drug classes, a number of promising antimalarial peptides of natural or synthetic origin have been reported previously and are listed in Table 2.

**Table 2.** Antimalarial peptides with their native source and mechanism of action.

Peptides	Sources	Mechanism of action	References
Apicidin**	*Fusarium pallidoroseum* (Fungal Metabolite)	Inhibits protozoan histone deacetylase (HAD)	[27]
Dermaseptin S4* (ALWMTLLKKVLKAAAKAALNAVLVGANA)	Frog skin	Inhibits the parasite’s ability to incorporate [3H] hypoxanthine	[46]
Dermaseptin S3* (ALWKNMLKGIGKLAGKAALGAVKKLVGAES)	Frog skin	Inhibits the parasite’s ability to incorporate [3H] hypoxanthine	[46]
Beauvericin	*Paecilo mycestenuipes* (Insect pathogenic fungus)	NA	[84]
Jasplakinolide*	*Jaspis* sp. (marine sponge)	Interfere with erythrocyte invasion by the merozoites	[76]
Dolastatin 10*	*Dolabella auricularia* (Sea hare)	Microtubule inhibitor	[39]
CEL-1000 (DGQEEKAGVVSTGLIGGG)**	b-chain of the human major histocompatibility complex class II molecule	Elicited Immune response	[22]
Hirsutellic acid A*	*Hirsutella* sp. BCC 1528 (Entomopathogenic fungus)	NA	[116]
Venturamide*	*Cyanobacterium oscillatoria*	NA	[68]
Antiamoebin I*	*Emericellopsis* poonensis (Fungus)	Inhibitors of mitochondrial activity	[82]
Efrapeptin*	*Tolypocladium niveum* (Fungus)	Inhibitors of mitochondrial activity	[82]
Zervamicin*	*Emericellopsis salmosynnemata* (Fungus)	Inhibitors of mitochondrial activity	[82]
Tyrothricin*	*Bacillus brevis* (Bacteria)	Exert its parasitic inhibition by rapid and selective lysis of infected erythrocytes	[98]
Isariins*	*Isaria* (Fungus)	Exact mechanism not known, inhibitory effect on the intra-erythrocytic growth of Plasmodium	[104]
Peptide IDR-1018 (Immune defence regulator)**	Host defence peptides (Synthetic)	Ability to modulate inflammatory responses	[1]
Benzyloxycarbonyl Z-Phe-Arg-CH2F*	Synthetic	Inhibit haemoglobin degradation by acting on cysteine proteinase	[103]
Phe-Orn-Phe-Orn*	Synthetic	NA	[93]
Lys-Phe-Phe-Orn*	Synthetic	NA	[93]

* *In vitro* study.

** *In vitro* and *vivo* study.

NA: Not available.

### Antimalarials under development

For diseases like malaria, there is an urgent need for active drug candidates that combat developing resistance mechanisms of *Plasmodium*. To address this concern, various lead molecules and vaccines are in the pipeline of drug discovery that can target *Plasmodium* at its different life cycle stages, Pre-erythrocytic (liver stage), Erythrocytic (blood stage), or Post-erythrocytic (gametocytic stage) to prevent relapses and transmission. They are listed in Table 3.

**Table 3.** Antimalarial compounds and clinical trial phases.

Product name	Clinical trial phase	Target stage/site
Malaria vaccine 257049	Phase III	Pre-erythrocytic stages, Erythrocytic stages
FMP011/AS01B	Phase I/II trial	Pre-erythrocytic stages, Erythrocytic stages
FMP2.1/AS02A	Phase II	Pre-erythrocytic stages, Erythrocytic stages
FMP1/AS02A	Phase II completed	Pre-erythrocytic stages, Erythrocytic stages
RTS, S/AS02D	Phase II completed	Pre-erythrocytic stages, Erythrocytic stages
Recombinant hybrid GMZ 2 [GLURP + MSP 3]	Phase I	Erythrocytic stage
Malaria vaccine candidates (VAC045) ChAd63-MVA CS ChAd63-MVA ME-TRAP	Phase I	Pre-erythrocytic stage
	Phase II completed	
*Plasmodium falciparum* malaria protein 010 (FMP010)	Phase I completed	Erythrocytic stage
Falciparum malaria protein (FMP012), E. Coli-expressed PfCelTOS	Phase I completed	Pre-erythrocytic stage
Peptides MSP3 long synthetic peptide 30 micrograms of MSP3 LSP	Phase II	Erythrocytic stage
Protease inhibitors Lopinavir/ritonavir Efavirenz Zidovudine Lamivudine	Phase III completed	Erythrocytic stage
OZ439 (second generation endoperoxide)	Phase II	Erythrocytic stage
KAE609	Phase II completed	Post-erythrocytic stage
NITD609 (spiroindolone class, plant Product)	Phase II	Post-erythrocytic stage
Ferroquine (SSR-97193, FQ),	Phase II completed	Erythrocytic stage
Trioxaquine SAR116242	Preclinical	Erythrocytic stage
MK4815	Preclinical	Erythrocytic stage
GNF156; an imidazolopiperazine	Phase II	Pre-erythrocytic, Erythrocytic Post-erythrocytic stage
DSM265 selective triazolopyrimidine-based inhibitor (first compound to target DHODH)	Phase I	Erythrocytic stage
P218 inhibitor of DHFR	Phase I	Erythrocytic stage
CDRI97/98	Phase I	Erythrocytic stage
CDRI9778	Phase I	Erythrocytic stage
Methylene blue	Phase II	Erythrocytic stage
*Argemone mexicana*	Phase II	NA
Rapid diagnostic test for malaria	Phase IV completed	Based on Immuno-detection of HRP2, pLDH

Source: http://www.clinicaltrials.gov/

## Conclusion

Currently, the biggest concern all over the globe is to treat patients with safe and effective medications and to avoid the emergence of drug-resistant malaria parasites. However, the emergence of vector resistance to widely used insecticides and parasite resistance to first-line drugs including artemisinin combination therapy has resulted in a rise in malaria incidence in many endemic areas, which has called for development of new therapeutic and technology approaches to combat the disease and impede drug resistance. However, more progress and better understanding in terms of scientific research and innovation is needed to develop these novel technologies as tools to reduce the occurrence of malaria.

## Conflict of interest

Authors have no conflict of interest.
